# Study on Poultry Pose Estimation Based on Multi-Parts Detection

**DOI:** 10.3390/ani12101322

**Published:** 2022-05-22

**Authors:** Cheng Fang, Haikun Zheng, Jikang Yang, Hongfeng Deng, Tiemin Zhang

**Affiliations:** 1 College of Engineering, South China Agricultural University, 483 Wushan Road, Guangzhou 510642, China; gu5457111@gmail.com (C.F.); haikun0619@gmail.com (H.Z.); yjkscau@gmail.com (J.Y.); redmaple-deng@outlook.com (H.D.); 2 National Engineering Research Center for Breeding Swine Industry, Guangzhou 510642, China; 3 Guangdong Laboratory for Lingnan Modern Agriculture, Guangzhou 510642, China

**Keywords:** pose estimation, broiler chicken, deep learning, object detection, precision agriculture

## Abstract

**Simple Summary:**

Poultry farming is an important part of China’s agriculture system. The automatic estimation of poultry posture can help to analyze the movement, behavior, and even health of poultry. In this study, a poultry pose-estimation system was designed, which realized the automatic pose estimation of a single broiler chicken using a multi-part detection method. The experimental results show that this method can obtain better pose-estimation results for a single broiler chicken with respect to precision, recall, and *F*1 score. The pose-estimation system designed in this study provides a new means to provide help for poultry pose/behavior researchers in the future.

**Abstract:**

Poultry pose estimation is a prerequisite for evaluating abnormal behavior and disease prediction in poultry. Accurate pose-estimation enables poultry producers to better manage their poultry. Because chickens are group-fed, how to achieve automatic poultry pose recognition has become a problematic point for accurate monitoring in large-scale farms. To this end, based on computer vision technology, this paper uses a deep neural network (DNN) technique to estimate the posture of a single broiler chicken. This method compared the pose detection results with the Single Shot MultiBox Detector (SSD) algorithm, You Only Look Once (YOLOV3) algorithm, RetinaNet algorithm, and Faster_R-CNN algorithm. Preliminary tests show that the method proposed in this paper achieves a 0.0128 standard deviation of precision and 0.9218 ± 0.0048 of confidence (95%) and a 0.0266 standard deviation of recall and 0.8996 ± 0.0099 of confidence (95%). By successfully estimating the pose of broiler chickens, it is possible to facilitate the detection of abnormal behavior of poultry. Furthermore, the method can be further improved to increase the overall success rate of verification.

## 1. Introduction

Poultry farming is an important part of China’s agriculture. With agricultural researchers’ growing interest in precision agriculture and intelligent agriculture, the application of computer vision technology in the agricultural production management system is increasing [1,2,3,4]. In recent years, computer vision technology was proven by scholars to be an efficient method of posture estimation and behavior analysis [5,6]. Indeed, the automatic application of computer vision technology has provided a noticeable improvement in agricultural production management [7,8].

Zhuang et al. used image-processing methods to identify the skeleton posture of yellow-feathered broiler chickens [9]. Khan et al. used a dense stacked hourglass network to estimate the posture of pigs through RGB images acquired by a head-mounted Kinect camera. The algorithm adopted a bottom-up approach, labeled nine key points, and analyzed the pose behavior in an actual farm environment [10]. Fuentes et al. used the cattle behavior recognition framework based on a deep neural network to conduct behavior recognition of the context-time information of the cattle in the video, which can identify up to 15 activities at different levels of the cattle [11]. Riekert et al. used deep learning to automatically detect the posture and position of pigs using a 2D camera. The algorithm’s detection accuracy of posture and position reached 80.2% [12].

In multi-part detection of livestock, Huang et al. used the improved SSD algorithm to detect multiple parts of the rear-view image of cows and realized automatic cow body condition scoring (BCS) with 98.46% classification accuracy and 89.63% positioning accuracy of the algorithm [13]. Hu et al. classified cows by the fusion of features of multiple parts (head, trunk, and legs), and the recognition accuracy reached 98.36% [14]. Marsot et al. identified pig faces by detecting the eyes, nose, and other parts of 10 pigs, and the detection accuracy for a total of 320 test pictures reached 83% [15]. Salau et al. used k-nearest neighbor and neural network to classify the head, rump, back, legs, and udders of cattle, where the Hamming loss of the k-nearest neighbor classification was between 0.007 and 0.027, and the Hamming loss of the neural network was between 0.045 and 0.079 [16]. Wutke et al. automatically tracked and analyzed abnormal behaviors between pigs, such as tail-biting and ear-biting, using a combination of a pose-estimation algorithm to detect key points in multiple body parts of pigs and a Kalman filter, achieving 94.2% sensitivity, 95.4% accuracy, 95.1% F1 score, and 94.4% MOTA score [17].

With the development of deep-learning technology, there is a growing body of research using deep learning to estimate the posture of animals. For example, Mathis et al. successfully used CNN to develop a Deeplabcut framework that can analyze human and animal posture [18]. Pereira et al. developed the LEAP pose-estimation software to analyze animal postures and validated its performance by using fruit fly images [19]. Raman et al. conducted feature localization and spatio-temporal analysis of dog movement and posture through sequence CNN [20]. 

Based on the powerful tool of deep learning, this paper proposes a pose-estimation algorithm based on deep neural networks for broiler chickens. This paper aims to realize an automatic pose estimation of broiler chickens and precise monitoring of large-scale poultry farms, and the potential application of this method in poultry behavior analysis is further analyzed by comparing the ability of this method and four other commonly used pose-estimation algorithms to estimate the posture of individual chickens in the flock. The automatic estimation of poultry posture can help subsequent poultry researchers to analyze movement, behavior, and even the health of poultry.

## 2. Materials and Methods

### 2.1. Experimental Environment

**Figure 1:**
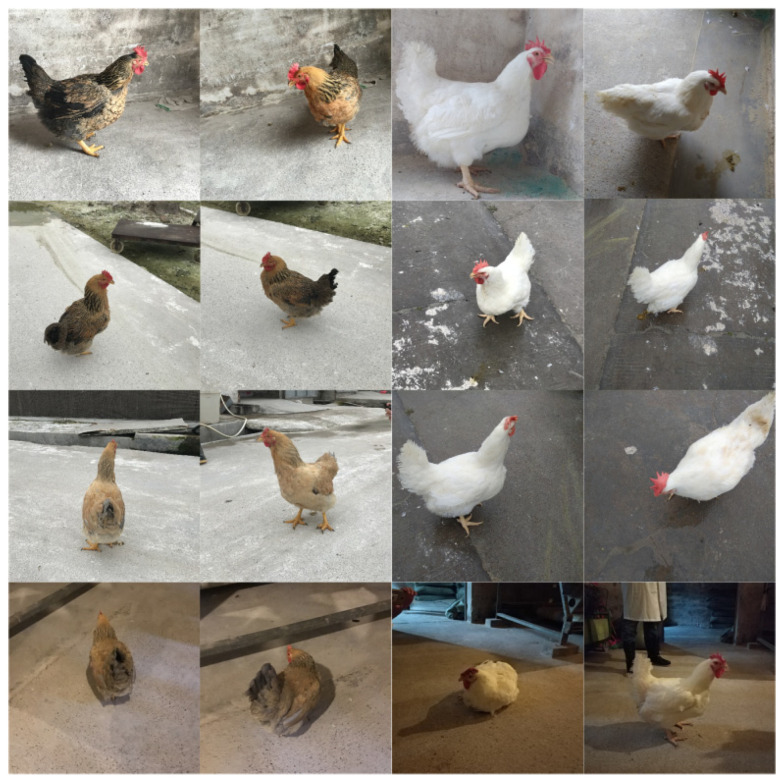
Experimental environment and test object.

The experimental environment is shown in Figure 1. The experiment in this paper was conducted in a poultry farm in Gaoming District, Foshan City, Guangdong Province, PR China. This poultry farm has two kinds of broilers, K90 and white recessive rock chickens (WRRC), both of which are common in Guangdong, so we studied their posture estimation in the research. Both the K90s and WRRCs were between 40 and 70 weeks old and were breeding birds, not for consumption. The image-acquisition system consisted of an HD camera (Logitech C922 Charge-Coupled Device camera) and a computer. The camera had a resolution of 1920 × 1080 pixels and took pictures of broiler chickens from multiple angles. Data were collected indoors and outdoors, with each collection time ranging from several seconds to several minutes, from 09:00 to 17:00 h. The indoor pen (4 m × 3 m) used both the natural and artificial photoperiod, while the outdoor one used only the natural photoperiod. The outdoor pen (6 m × 6 m) had enough space to allow the broilers free movement. The images collected by the camera were transmitted to the computer through a USB port for further processing. The experiment used a computer with a six-core processor, 2.4 GHz per core, 16 GB of RAM, a Windows 10 operating system, and a GTX 1060 6 G graphics card. 

**Figure 2:**
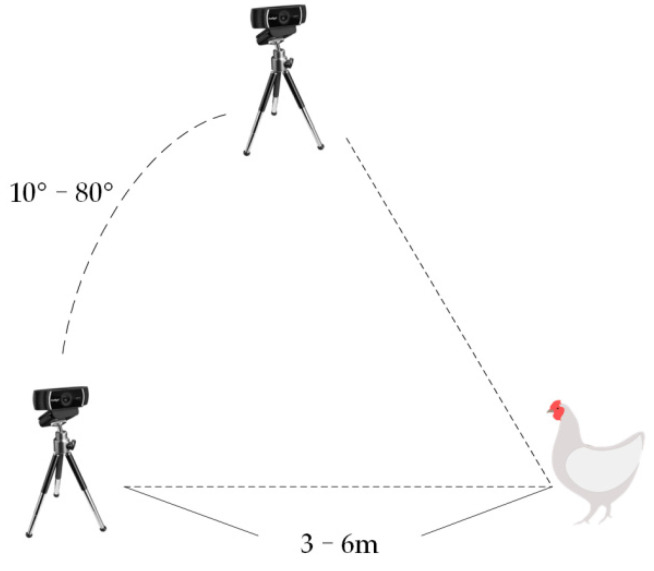
Photograph schematic.

The schematic is shown in Figure 2. The camera was 3–6 m away from the chicken at angles of 10 degrees to 80 degrees.

### 2.2. Data Processing and Labelling

**Figure 3:**
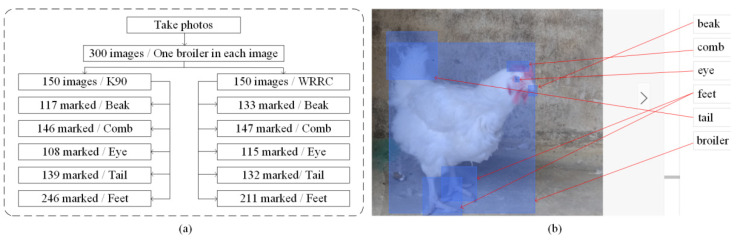
Datasets and marked examples: (**a**) Data partitioning; (**b**) Marked image as ground truth.

The collected data were screened, and any abnormal data caused by unexpected vibrations were eliminated. To reduce the memory consumption of GPU during training, all images collected were preprocessed by OpenCV (ver. 3.6.0) and the resolution adjusted to 512 × 512 pixels. The photos in the data set were manually labeled using EasyDL software to be used for subsequent pose estimation of a single chicken. The processed data set is shown in Figure 3.

The chicken dataset includes 300 images of broilers: 150 K90 broilers and 150 WRRC broilers. The K90 set contains 117 marked beaks, 146 marked combs, 108 marked eyes, 139 marked tails, and 246 marked feet. The WRRC set contains 133 marked beaks, 147 marked combs, 115 marked eyes, 132 marked tails, and 211 marked feet.

### 2.3. Algorithm Framework and Implementation Steps

**Figure 4:**
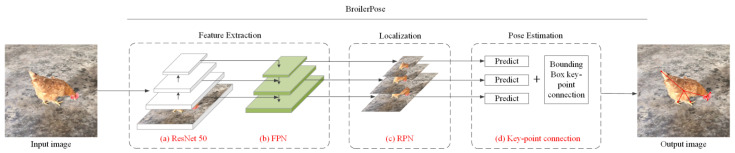
BroilerPose pose-estimation algorithm architecture: (**a**) The pre-trained ResNet-50; (**b**) FPN architecture, which includes a classify and a regression network; (**c**) RPN; (**d**) Key-point connection, output the broiler’s posture.

In this study, the algorithms were written in standard Python language. Figure 4 shows the flow of the BroilerPose pose-estimation algorithm, which references the Retinanet algorithm and consists of two steps [21]. The first step was to locate the target and the second step was to categorize the goals. A 50-layer residual network (ResNet-50) and a feature pyramid network (FPN) were used to construct the backbone to extract features from the image. The ResNet-50 was a bottom-up convolution network [22]. With the higher stage of convolution, the size of the resultant maps became smaller, and a higher level of semantics was retained. The FPN was a top-down convolution network. The lower-level feature layer in FPN was the combination of the higher-level feature layer and a corresponding ResNet-50 feature layer. The ResNet-50-FPN structure facilitated the extraction of both higher and lower-level relations [23]. Finally, the candidate frame was located and extracted through the Region Proposal Network (RPN), and the key points of the candidate frame were obtained and connected. Finally, the posture of the chicken was obtained.

After the broiler chicken pictures passed through the BroilerPose network structure, the results of six different categories were output. These were the bounding box (Bbox) of the broiler, beak, comb, eye, tail, and feet as $B_{\alpha }\left(x_{\alpha },y_{\alpha },x_{\alpha }+w_{\alpha },h_{\alpha }\right),\alpha \in \left[1,6\right]$. $\left(x_{\alpha },y_{\alpha }\right)$ was the coordinate of the upper left corner of the Bbox, while $\left(x_{\alpha }+w_{\alpha },y_{\alpha }+h_{\alpha }\right)$ was the coordinate point of the lower right corner of the Bbox. $w_{\alpha }$ and $h_{\alpha }$ were the width and height of the Bbox, respectively.

After obtaining the six Bbox categories, we output the central point of each Bbox as the key-point of the broiler chicken body part. The key-point was $K_{i}\left(X_{i},Y_{i}\right),i\in \left[1,8\right]$. $X_{i}$ and $Y_{i}$ are shown in Equation (1):

(1) $$\{\begin{array}{c} X_{i}=x_{\alpha }+\frac{1}{2}w_{\alpha }\\ Y_{i}=y_{\alpha }+\frac{1}{2}h_{\alpha } \end{array}$$

We then built the broiler chicken key-point connection algorithm, as shown in Table 1.

As broiler chickens exist in various postures, there may be situations where the Bbox cannot be detected. When the Bbox was not recognized, we did not connect the key-point.

### 2.4. Algorithm Training

The training code was completed under the Keras deep-learning framework. From the collected data, we established the data set of broiler chickens described in Section 2.2 and randomly mixed the video of the data set. The size of the image used for algorithm input was 512 × 512 pixels. The ratio of the training set to the test set was 9:1. The whole training was iterated 1000 times using Stochastic Gradient Descent (SGD) as the network optimizer. SGD updated the parameters through each iteration to speed up the training [24]. The initial learning rate was set at 0.02.

### 2.5. Evaluation Metrics

After the detectors were trained, the testing set was used for evaluating. To determine whether every part had been correctly recognized, the intersection over union for each predicted part was computed using the area of overlap and union (Equation (2)):

(2) $$IoU=\frac{Area\;of\;Overlap}{Area\;of\;Union}$$

An IoU greater than 0.5 means the detectors detected the part of broiler chicken correctly.

Precision, recall, mean average precision (mAP), and *F*1-score for detecting each part of the broiler chickens in the images were calculated using Equations (3)–(5). Precision is the ratio of true detection in all cases. Recall is the ratio of true detection in all manually labeled cases. *F*1-score is the harmonic mean of precision and recall and a balancing metric for comprehensively evaluating false and missing cases:

(3) $$P=\frac{TP}{TP+FP}$$

(4) $$R=\frac{TP}{TP+FN}$$

(5) $$F1=\frac{2\cdot P\cdot R}{P+R}=\frac{2TP}{2TP+FP+FN}$$

where TP is true positive; FP is false positive; FN is a false negative; and TN is a true negative.

Average precision (*AP*) is defined as the area under the precision–recall curve and expressed as the mean precision at a set of 11 equally spaced recall levels [0, 0.1, …, 1] [25]. The precision–recall curve is produced according to the predicted confidence level. The calculation of the *AP* is shown in Equation (6):

(6) $$AP_{point=11}={\frac{1}{11}}_{\tilde{k}\in \left\{0,0.1,\ldots ,1\right\}}\sum max_{\tilde{k}\geq k}P\left(\tilde{k}\right)$$

where $\tilde{k}$ is eleven equally spaced recall levels from 0 to 1. The maximum measured precision within a wiggle piece of the precision–recall curve was also used.

The mean average precision (mAP) is the average value of *AP* obtained for six different categories. 

## 3. Results

### 3.1. Effects of Different Detection Methods

**Figure 5:**
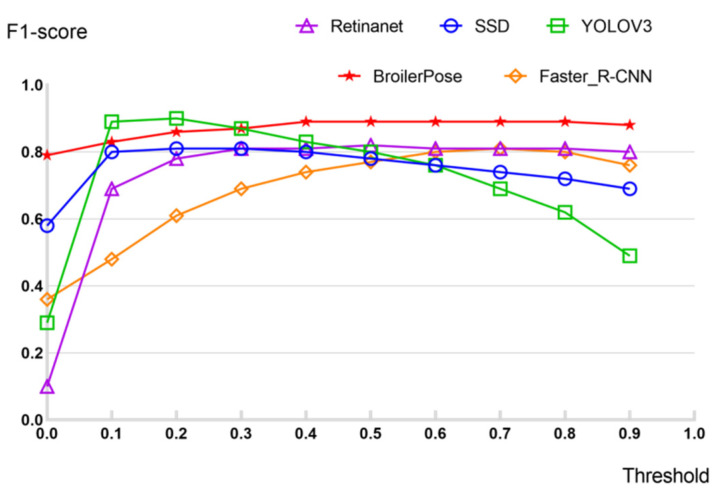
*F*1-score for different algorithms, including BroilerPose algorithm, YOLOV3 algorithm, RetinaNet algorithm, SSD algorithm, and Faster_R-CNN algorithm. The higher the *F*1-score, the better the detection effect.

In the paper, five different methods were used to test the broiler chicken set, namely, BroilerPose, SSD, YOLOV3, RetinaNet, and Faster_R-CNN. Figure 5 shows the *F*1-Score of five different algorithms with different thresholds.

By comparing the test results of five pose-estimation algorithms, the BroilerPose algorithm proposed in the paper reaches 0.89 in *F*1-score when the threshold value is equal to 0.5. The RetinaNet algorithm achieves an *F*1-score of 0.82. It is followed by the YOLOV3 algorithm (*F*1-score = 0.80), the SSD algorithm (*F*1-score = 0.78), and Faster_R-CNN (*F*1-score = 0.77). We then calculated the overall AP of each algorithm and the mAP of individual parts.

**Figure 6:**
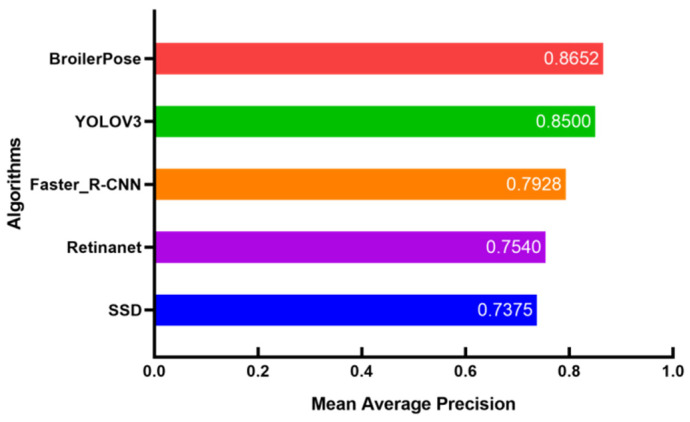
The performance of different algorithms in mAP.

Figure 6 shows the scores for different algorithms on the mAP, which can show the degree of the test model in all categories.

The mAP of the BroilerPose algorithm is 0.8652, the YOLOV3 algorithm is 0.8500, the Faster_R-CNN algorithm is 0.7928, the RetinaNet algorithm is 0.7540, and the SSD algorithm is 0.7375. Meanwhile, the training effects of different was shown in Table 2.

The results show that in the broiler Bbox, the recognition performance of the five algorithms reached more than 99%, among which the SSD algorithm was the highest, reaching 99.9%. In the beak and tail detection frame, YOLOV3 achieved the best results, reaching 77.4% and 90.4%, respectively. The BroilerPose algorithm proposed in this paper achieved the best results in the comb, eye, and feet (83.7%, 79.0%, and 90.2%, respectively). The precision and recall of various algorithms was shown in Table 3.

The precision index from high to low is 93.3% (YOLOV3), 91.9% (BroilerPose), 88.1% (RetinaNet), 84.0% (Faster_R-CNN), and 83.8% (SSD).

The recall index from high to low is 86.5% (BroilerPose), 85.0% (YOLOV3), 79.3% (Faster_R-CNN), 75.4% (RetinaNet), and 73.7% (SSD).

### 3.2. Comparison of Posture of Different Models

**Figure 7:**
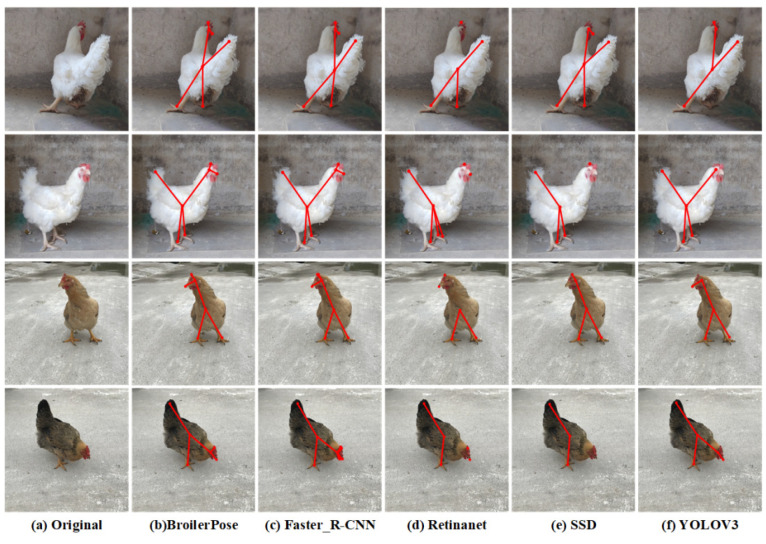
Partial results of posture comparison of broiler chickens.

To verify the pose-estimation ability of the algorithm, we selected pictures of broiler chickens from different angles for pose comparison. Figure 7 shows the partial results of the posture comparison of some broiler chickens.

The results of the test set were statistically analyzed. For the entire test set, the standard deviation of precision was 0.0128, the value of confidence (95%) was 0.9218 ± 0.0048, the standard deviation of recall was 0.0266, and the value of confidence (95%) was 0.8996 ± 0.0099. For K90, the standard deviation of precision was 0.0096, the value of confidence (95%) was 0.9255 ± 0.0053, the standard deviation of recall was 0.0267, and the value of confidence (95%) was 0.8888±0.0148. For WRRCs, the standard deviation of precision was 0.0147, the value of confidence (95%) was 0.9181 ± 0.0081, the standard deviation of recall was 0.0225, and the value of confidence (95%) was 0.9105 ± 0.0124. Table 4 shows the results for two types of broilers indoors and outdoors.

## 4. Discussion

The comparison between the BroilerPose pose-estimation algorithm and the other four algorithms shows that the BroilerPose pose-estimation algorithm demonstrates better pose-estimation performance. 

Faster_R-CNN: The pyramid model can be used to solve the problem of the RCNN clipping scale change [26]. The attention mechanism in Natural Language Processing (NLP) is used for reference. The classification of regions of interest improves the speed of candidate box collection and has better detection for small objects; see Figure 7c.

YOLOV3 completes object positioning and classification together and returns to the bounding box’s position and category at an output level [27]. Because there is no regional sampling, it has a good performance in global information, but it has a poor performance in small-scale information such as detecting the eye and comb; see Figure 7f. 

SSD is an algorithm that uses a DNN to detect and classify objects in an image simultaneously. The algorithm generates a set of default boxes with different aspect ratios and sizes and matches the default boxes with the real boxes to predict the confidence of object identification and position offset [28]. Like YOLOV3, SSD has poor performance in small-scale detection; see Figure 7e.

RetinaNet proposed a new loss function, Focal Loss, which can solve the problem of unbalanced positive and negative samples in target detection. However, in some cases where the detection target is relatively small, the recognition effect is not good; see Figure 7d.

However, by setting different IoU thresholds in the R-CNN of the network, BroilerPose performs well at small target detection; see Figure 7b.

For the test set, the standard deviation of precision between K90 and WRRC was 0.0096 and 0.0147; both test results show stability in precision. Meanwhile, both broilers had confidences (95%) of precision of more than 0.9, so the algorithm can properly work in both cases.

The BroilerPose pose-estimation algorithm performs well in mAP and *F*1-Score. After the accurate pose estimation, we can lay a foundation for follow-up behavior analysis. Through the analysis of the test data, the algorithm proposed in this paper has a good effect on the accuracy and recall rate of two different breeders. Furthermore, we can combine the tracking algorithm and BroilerPose pose-estimation algorithm to carry out continuous pose estimation and even behavioral analysis for single broiler chickens [29,30] to judge the movement state, health state, and welfare of broiler chickens.

## 5. Conclusions

In this paper, a pose-estimation algorithm based on DNN is proposed to estimate the pose of a single broiler chicken. By comparing this algorithm with other pose-estimation algorithms, the results shows that the precision and recall of this algorithm for a single broiler chicken pose estimation is 91.9% and 86.5%, respectively. The test set shows stability in the precision between K90 and WRRC, and both broilers had confidences (95%) of more than 0.9. In conclusion, the proposed method can recognize the posture of individual chickens, which is helpful for poultry researchers and accurate detection in large-scale farms. 

Our method can estimate the pose of a single broiler chicken from multiple angles. In the case of multiple broiler chickens, however, there are all sorts of problems. Therefore, for future work, we hope to conduct more studies on poultry pose estimation through in-depth study.

## Figures and Tables

**Table 1:** Key-point connection combination.

Part	Key-Point	Combination
Broiler	K _1_	(*K*_1_, *K*_4_)
Beak	K _2_	(*K*_2_, *K*_4_)
Comb	K _3_	(*K*_3_, *K*_4_)
Eye_left	K _4_	(*K*_4_, *K*_4_)
Eye_right	K _5_	(*K*_5_, *K*_1_)
Tail	K _6_	(*K*_6_, *K*_1_)
Foot_left	K _7_	(*K*_7_, *K*_1_)
Foot_right	K _8_	(*K*_8_, *K*_1_)

**Table 2:** Comparison of training effects of different algorithms.

Bbox	Algorithms				
	BroilerPose	YOLOV3	Faster_R-CNN	RetinaNet	SSD
Broiler	0.997	0.998	0.994	0.998	0.999 ^1^
Beak	0.772	0.774 ^1^	0.65	0.641	0.563
Comb	0.837 ^1^	0.756	0.651	0.785	0.772
Eye	0.790 ^1^	0.768	0.74	0.728	0.734
Tail	0.893	0.904 ^1^	0.873	0.891	0.901
Feet	0.902 ^1^	0.9	0.849	0.897	0.816

^1^ This means that this value has the highest score in the corresponding Bbox.

**Table 3:** Precision and recall of various algorithms.

	BroilerPose	YOLOV3	Faster_R-CNN	RetinaNet	SSD
Precision	0.919	0.933 ^1^	0.840	0.881	0.838
Recall	0.865 ^1^	0.850	0.793	0.754	0.737

^1^ This means that this value has the highest score in the corresponding index.

**Table 4:** Precision and recall of various situations.

		K90	K90 (Indoor)	K90 (Outdoor)	WRRC	WRRC (Indoor)	WRRC (Outdoor)	All
Standard deviation	Precision	0.0096	0.0106	0.0092	0.0147	0.0081	0.011	0.0128
	Recall	0.0267	0.0375	0.0183	0.0225	0.0173	0.0226	0.0266
Confidence (95%)	Precision	0.9255 ± 0.0053	-	-	0.9181 ± 0.0081	-	-	0.9218 ± 0.0048
	Recall	0.8888 ± 0.0148	-	-	0.9105 ± 0.0124	-	-	0.8996 ± 0.0099

## Data Availability

Data sharing is not applicable to this article.
